# Differences and Similarities among Parotoid Macrogland Secretions in South American Toads: A Preliminary Biochemical Delineation

**DOI:** 10.1155/2013/937407

**Published:** 2013-04-30

**Authors:** Juliana Mozer Sciani, Cláudia Blanes Angeli, Marta M. Antoniazzi, Carlos Jared, Daniel Carvalho Pimenta

**Affiliations:** ^1^Laboratório de Bioquímica e Biofísica, Instituto Butantan, Av. Vital Brasil 1500, 05503-900 São Paulo, SP, Brazil; ^2^Departamento de Parasitologia, Instituto de Ciências Biomédicas da Universidade de São Paulo, Av. Prof. Lineu Prestes 2415, 05508-000 São Paulo, SP, Brazil; ^3^Laboratório de Biologia Celular, Instituto Butantan, Av. Vital Brasil 1500, 05503-900 São Paulo, SP, Brazil

## Abstract

Amphibians are known by cutaneous glands, spread over the skin, containing toxins (proteins, peptides, biogenic amines, steroidal bufadienolides, and alkaloids) used as chemical defense against predators and microbial infection. Toads are characterized by the presence of parotoid macroglands. The common toads have lately been divided into two genera: *Bufo* (Europe, Asia, and Africa) and *Rhinella* (South America). Basal *Rhaebo* genus is exclusively of Central America and Amazon region. Although *Rhinella* and *Rhaebo* are related, species may share differences due to the diversity of environments that they live in. In this work, we have performed a biochemical characterization of the components of the poison of eight *Rhinella* species and one *Rhaebo* by means of RP-HPLC with either UV or MS detection and by SDS-PAGE, in order to verify whether phylogenetic and biological differences, such as habitat, diet, and defensive strategies, between them may also be reflected in poison composition. Although some components were common among the secretions, we were able to identify exclusive molecules to some species. The fact that closely related animals living in different habitats secrete different molecules into the skin is an indication that biological features, and not only evolution, seem to directly influence the skin secretion composition.

## 1. Introduction

Amphibians, in general, are characterized by the presence of cutaneous glands spread over the whole body skin, basically of two different types: (i) mucous, generally associated to maintenance of humidity and cutaneous respiration, and (ii) granular glands, generally associated with chemical defense against predators and/or microbial infection [1, 2]. The product secreted by such glands consists of a wide variety of chemical compounds including proteins, peptides, biogenic amines, toxic steroidal bufadienolides, toxic samandarine alkaloids, and indolic pseudophrynamine alkaloids, depending on the species [3, 4]. In comparison to the enormous range of molecules secreted by these animals [5–8], studies evaluating the biochemical and pharmacological activities of these compounds are still scarce [9, 10].

In many amphibians, the granular (or poison) glands can be grouped and enlarged in special regions forming macroglands, which have evolved as protection against the attack of specific predators. This is the case of toads, characterized by the presence of a pair of parotoid macroglands, strategically located behind the eyes to give protection by poison release in the form of jets in case of frontal attacks [2].

Bufonid parotoid macroglands, largely known by their toxic secretions, contain bufotoxins that, in contact with the oral mucosa of the predators, may present cardiac glycoside-like activity, increasing the contractile force of the heart [11]. The poison can also exert a marked effect as a local anaesthetic [12]. In the case of a predator biting a toad, the poison released from the parotoid makes contact with the oral mucosa reaching the blood stream. It causes intense salivation and excitation, paralysis, trembling, and convulsions, very often leading to death [13–17]. In addition to these organismic effects, experimental essays with parotoid secretions revealed antimicrobial activities [9, 18].

 Common toads, until recently, used to be all grouped in the genus *Bufo*. In South America they are now divided into two genera: *Rhinella* and *Rhaebo* [19]. With this new classification, genus *Bufo* is now basically restricted to species in Europe, Asia, and Africa [20]. While the genus *Rhinella* comprises eighty-six species spread over all types of biomass, genus *Rhaebo* comprises only nine species, distributed exclusively in the equatorial region, from Honduras to Colombia, going through Ecuador, Venezuela, Guianas, Peru, Bolivia, and Brazil [20–22].

 Although the species of genera *Rhinella* and *Rhaebo* are all related, they may have profound differences, due to the diversity of environments and microhabitats they live in. For example, while *Rhinella jimi* is a large endemic species inhabiting the semiarid Brazilian biome (the Caatinga) in Northeastern Brazil, the smaller *Rhinella major* is native from the Amazonian rainforest floor. Also, behaviors can largely vary, as the unique defense strategy of *Rhaebo guttatus,* that is, being able to actively and voluntarily squirt poison from its parotoid glands, an unexpected feature that was until recently considered to be an ancient myth [23].

 According to the classification of Pramuk (2006), South American toads can be divided into six great groups of species. Four of them have representatives in Brazil: *R. crucifer* group, *R. guttatus* group, *R. margaritifera* group, and *R. marina* group.

 Preliminary biochemical and pharmacological studies have been conducted, indicating that, although the parotoid secretions from different species of toads show many similarities, they also present some peculiarities, which are the result of phylogenetic and biological differences in habitats, defensive strategies, and diet.

In an attempt to better understand these differences and similarities between (and among) genera *Rhaebo* and *Rhinella*, we have performed a biochemical characterization of the major components of parotoid secretion of eight species of *Rhinella* and one species of *Rhaebo*, belonging to the four great groups of toads found in Brazil, as classified by Pramuk [21].

## 2. Material and Methods

### 2.1. Reagents

All chemicals employed in this work were of analytical grade and were purchased from Sigma-Aldrich Co. (St. Louis, MO, USA) unless otherwise stated. 

### 2.2. Parotoid Secretion

Animal collection and housing were performed under appropriate IBAMA licenses. The parotoid secretion of specimens of *R. jimi*, *R. crucifer, R marina, R schneideri, R. icterica*, *R. guttatus*, *R. granulosa*, *R. major,* and *R. margaritifera* was obtained by manual compression of the parotoid macrogland. The extremely viscous secretion was collected in a centrifuge tube and stored at −20°C. At the moment of use, the secretion was extracted with 0.1% trifluoroacetic acid (TFA)/H_2_O. The solution was sonicated for 5 minutes and centrifuged at 5000 ×g for 5 minutes. The supernatant separated and further processed, as follows.

### 2.3. Biochemical Characterization

#### 2.3.1. RP-HPLC

The parotoid secretion of the toad species was analyzed by reversed phase high performance liquid chromatography (RP-HPLC) using a binary HPLC system (20A Prominence, Shimadzu Co., Japan). Ten microliters aliquots of the secretions were loaded in a C18 column (ACE C18, 5 *μ*m; 100 Å, 250 mm × 4.6 mm) in a two-solvent system: (A) TFA/H_2_O (1 : 1000) and (B) TFA/Acetonitrile (ACN)/H_2_O (1 : 900 : 100). The column was eluted at a constant flow rate of 1 mL·min^−1^ with a 0 to 100% gradient of solvent B over 20 min, after a 5 min isocratic elution with 0% B. The HPLC column eluates were monitored by a Shimadzu SPD-M20A PDA detector scanning from 200 to 500 nm (1 nm steps). Background subtraction was performed for chromatogram integration and processing.

#### 2.3.2. LC-MS

The mass spectrometry analyses were performed in an ESI mass spectrometry (LCQDuoTM, Thermo Finnigan, USA), equipped with a nanospray source and connected to a nano-HPLC system (UltiMate, LC Packings, Dionex, USA), coupled to a C18 nanocolumn. The samples were deposited into autosampler for MS analyses and 10 *μ*L sample aliquots were injected at a constant flow rate of 1 *μ*L·min^−1^. The spray voltage was kept at 1.8 kV, the capillary voltage at 46 V, the capillary temperature at 180°C, and the tube lens offset at −5 V. MS spectra were acquired under positive mode and collected in the 50–2000 *m*/*z* range. Instrument control, data acquisition, and data processing were performed with the Xcalibur Suite.

#### 2.3.3. SDS-PAGE

In order to compare the protein contents of the eight species of *Rhinella *and one species of* Rhaebo* parotoid gland secretion solution, a 12% polyacrylamide gel electrophoresis containing sodium dodecyl sulfate (SDS-PAGE) was performed, in reducing conditions, according to Laemmli [24]. 

## 3. Results

### 3.1. RP-HPLC and LC-MS

**Figure 1:**
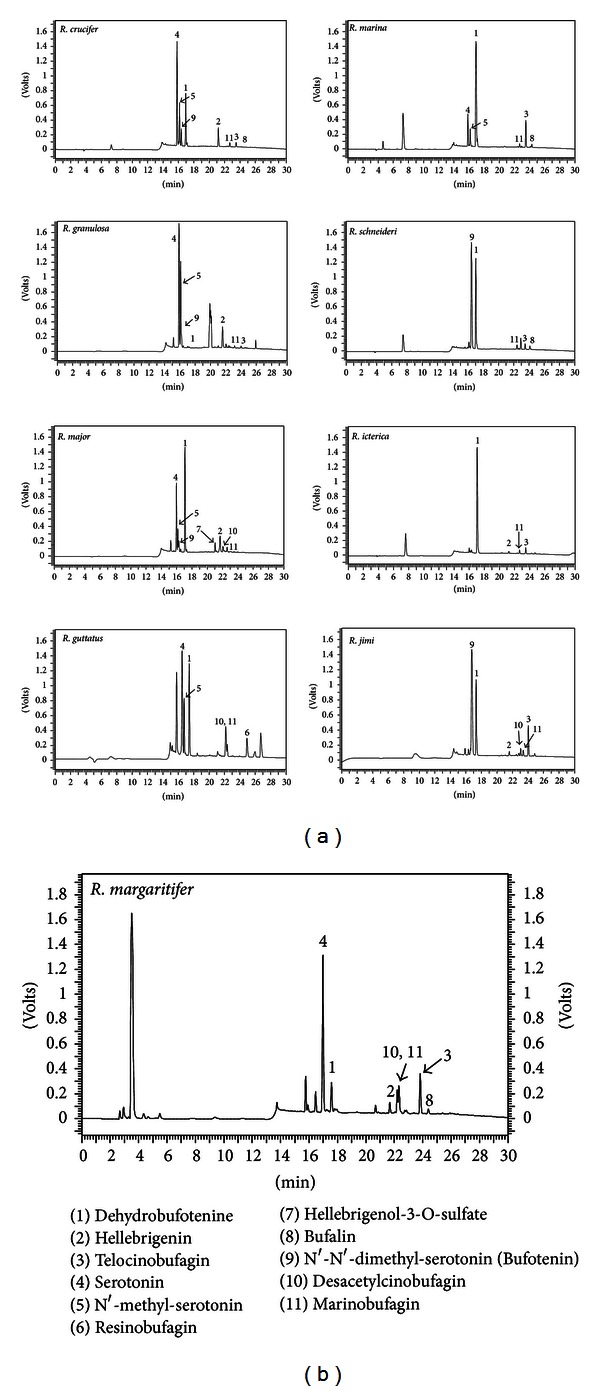
C18-RP-HPLC profiles (*λ* = 214 nm) of the parotoid secretion of the studied toads in this work with the identified molecules annotated.

 The aqueous skin secretion solutions of the nine toads (normalized at 1 mg·mL^−1^ according to their dry weight) were analyzed by C18 RP-HPLC, either by UV (Figure 1) or MS (data not shown) monitoring, according to Section 2.

**Figure 2:**
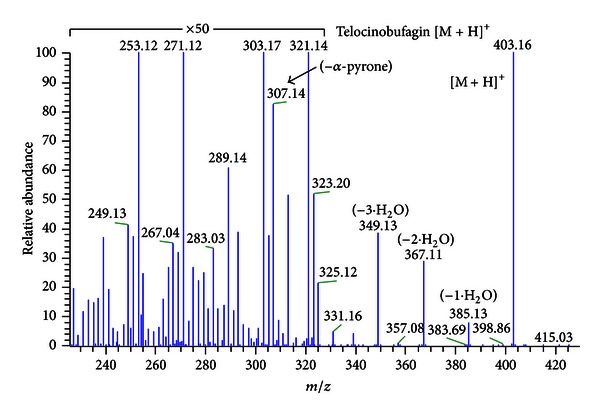
Off-line MS^2^ fragmentation pattern of the peak eluting at 24′ that was purified from *R. marina*, according to the profile depicted in Figure 1. The molecule could be identified as being telocinobufagin based on the daughter ions and published results.

**Figure 3:**
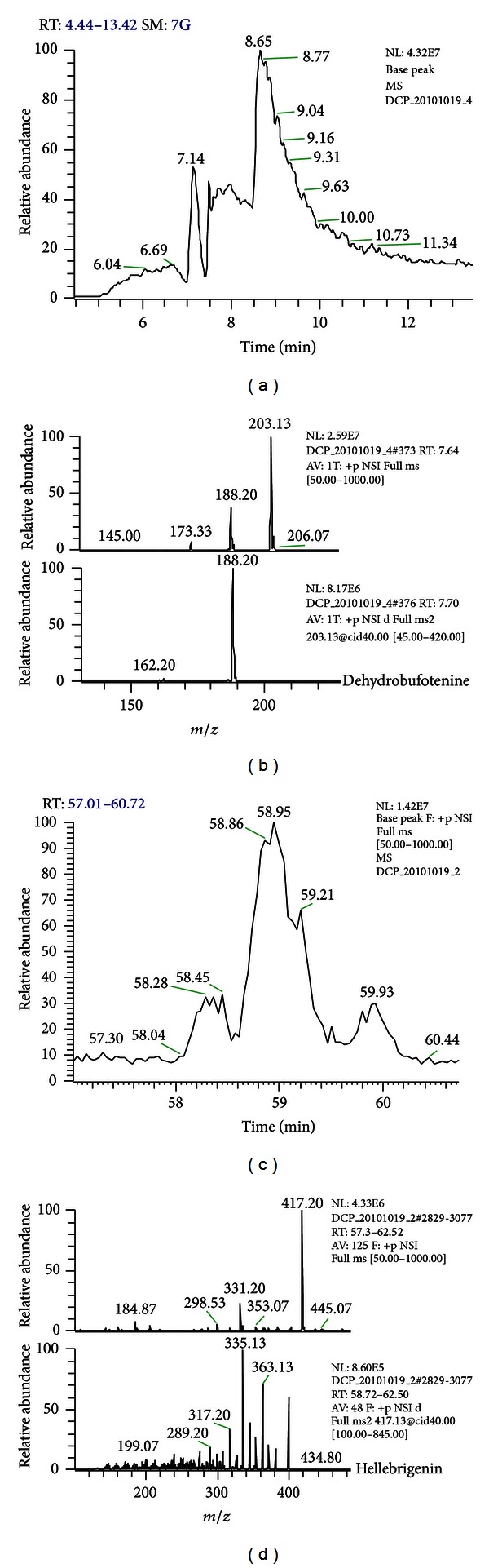
Detailed view of on-line LC-MS/MS analyses performed for the analyses of the skin secretion of *R. schneideri* ((a) zoomed TIC chromatogram and (b) MS and MS^2^ profiles) and *R. jimi* ((c) zoomed TIC chromatogram and (d) MS and MS^2^ profiles). The molecules could be identified as being dehydrobufotenine and hellebrigenin based on the daughter ions and published results.

The UV-monitored RP-HPLC analyses show that, in general, the 214 nm profiles (wavelength chosen due to the best signal to noise ratio and larger count of individual peaks) are similar: the major peaks lie in the middle of the gradient, followed by a group of more hydrophobic molecules, with about half the UV absorbance intensity, as shown in Figure 1. UV major peaks for the nine toad species were manually collected and submitted to ESI-IT/MS analyses for structural determination.  Figure 2 is an example of such analysis. One can observe that the peaks in the 15′ region were identified as being alkaloids and those at the 20′ (and over) as being the steroids, which is in accordance with their physicochemical characteristics and to previously reported data [6–8]. Moreover, these pieces of information could be confirmed by the LC-MS^2^ analyses performed, as exemplified in Figure 3.  Figure 3(a) contains a zoomed part of the TIC chromatogram for the RP-HPLC separation of *R. schneideri* venom (5–13′).  Figure 3(b) upper profile shows the MS profile of RT 7.6′ (in which some fragments can already be observed, probably generated at the cone), whereas in the lower profile the MS^2^ profile of *m/z* 203.13 can be observed. The overall analysis of these data, together with published spectra, made it possible to identify this molecule as being dehydrobufotenine. Figures 3(c) and 3(d) show the same rationale for the identification of hellebrigenin from *R. jimi*: Figure 3(c) is the zoomed TIC chromatogram for the RP-HPLC separation, and Figure 3(d) (upper) is the MS at RT 57–60′ and Figure 3(d) (lower) the MS^2^ of the *m/z* 417.13. The molecule was identified as hellebrigenin based on the fragmentation pattern as well as published data comparison.

**Figure 4:**
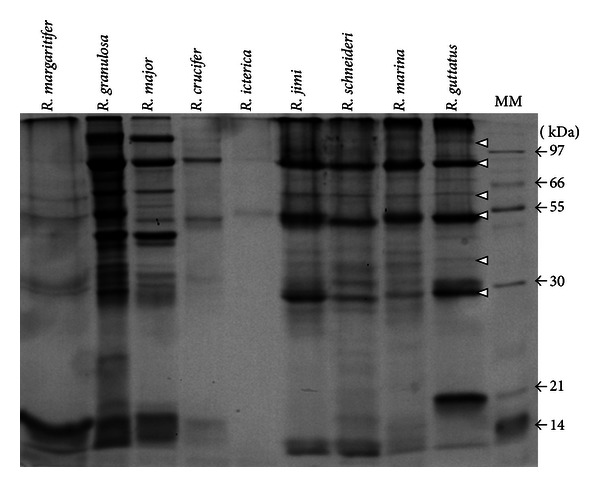
CBB stained SDS-PAGE of the parotoid secretion solutions analyzed in this work. MM: molecular mass marker. White arrow heads indicate the most constant proteins throughout the species.

*R. icterica* presented the least complex C18 RP-HPLC profile (as monitored by *λ* = 214 nm), in which one major peak clearly stands out (Figure 1). This peak was subjected to MS^2^ analyses and was demonstrated to be the known alkaloid dehydrobufotenine. The TIC profile for *R. icterica* (data not shown) and the SDS-PAGE (Figure 4) analysis also show that this skin secretion is, according to the employed techniques, the least biochemically complex among the analyzed secretions.

 Not surprisingly, *R. jimi* presented a similar profile, when compared to *R. icterica*. Both bufadienolide steroids could be identified, as well as dehydrobufotenine. The actual major peak could not be positively identified according to its MS and MS^2^ profiles, when compared to published data [5–8]. TIC chromatogram for this secretion, on the other hand (data not shown), shows that more molecules are present and were more able to ionize at the selected conditions than those for *R. icterica*. Although some of these molecules could be identified according to their MS and MS^2^ profiles, the poorer chromatographic resolution (Figures 3(a) and 3(c)) makes this profile less suitable for qualitative comparison. Nevertheless, once a parent ion is properly selected, MS and MS^2^ analyses allow unequivocal spectra interpretation (Figures 3(b) and 3(d)). 

*R. marina *and *R. schneideri*, toads from the same group of *R. jimi* and *R. icterica,* also had the parotoid secretion analyzed, in the same conditions. *R. schneideri *UV chromatogram was very close to *R. jimi* and *R. icterica*, as well as the TIC chromatogram and the identified molecules. *R. marina* has the same molecules as the others, except N′-N′-dimethyl-serotonin, but has alkaloids serotonin and N′-methyl-serotonin.

*R. guttatus* aqueous skin secretion solution presented more hydrophilic peaks, which include serotonin and N-methyl-serotonin, the same of *R. marina*. Dehydrobufotenine was also identified in this animal. Hydrophobic peaks were also present in the chromatogram, and one could be identified as being resibufogenin. The UV profile resembles *R. crucifer* group, because it presented the same main components. However, *R. guttatus* presented some unidentified peaks. 

*R. crucifer*, *R. granulosa,* and *R. major* skin secretion UV profiles showed the same two-group peak distribution, including dehydrobufotenine, which corresponds to the largest peak for *R. major*, the second largest peak for *R. crucifer* and, on the other hand, is a very small peak for *R. granulosa*. These observations were corroborated by the ESI-IT/MS analyses, in which the TIC chromatograms were even more related for *R. granulosa *and *R. major* and more resolved TIC chromatogram, similar to that of *R. icterica*, but containing much more peaks, especially at the more hydrophilic region, which is in accordance with the RP-HPLC profiles. Although the LC-MS profile of *R. crucifer* is different from the others, serotonin and N-methyl-serotonin were also identified in these animals, either in terms of retention time or by LC-MS and MS^2^ profiles. At the steroid region, both *R. granulosa* and *R. major* presented hellebrigenin and telocinobufagin. *R. granulosa* presented a unique group of peaks (at RT ~ 19′) which did not match any published MS/MS^2^ profile. On the other hand, *R. major* skin secretion exclusive feature was the presence of a sulfonated steroid (hellebrigenol-3-O-sulfate), and for *R. crucifer*, bufalin was the exclusive molecule. The UV profile of *R. margaritifer* showed that in terms of alkaloids, the hydrophilic peaks resemble *R. crucifer* group, and steroids resemble *R. marina* group. The LC-MS profile is related to *R. crucifer*.

### 3.2. SDS-PAGE

All animals presented a similar SDS-PAGE profile (Figure 4). Although the relative protein concentration seems to vary among the species, several proteins, according to their *M*_*r*_, seemed to be very constant (white arrow heads). *R. jimi*, *R. schneideri*, *R. marina,* and *R. guttatus* possess a virtually identical profile, except for one exclusive band being for *R. guttatus* (~20 kDa). *R. icterica, R. crucifer,* and *R. margaritifer* presented poor protein profiles, although the relative concentration of secretion was similar to other toads. The band resolution for some proteins, especially at the low *M*_*r*_ region, is poor for *R. margaritifer*.

## 4. Discussion

In order to perform an unbiased comparison, we have chosen nine toad species coming from three different habitats and belonging, according to Pramuk [21], to the five different groups found in Brazil, as presented in Table 1.

It is our understanding that any possible key variation would be neutralized, for the chosen animals encompass related species sharing the same habitat (e.g., *R. jimi *and *R. granulosa*), related species geographically separated (e.g., *R. icterica, R. jimi, *and *R. marina*), different species sharing the same habitat (e.g., *R. guttatus, R. marina,* and *R. major*), and one more basal species (*R. guttatus*).

Taking into account the criteria displayed above, our data show that there is an evident dissociation between the alkaloids and steroids secretion pattern into the skin of the toads and their habitat (Figure 1 and Table 1); for example, animals leaving in completely different habitats (such as the rain forest on in the semiarid) will produce a rather similar RP-HPLC profile. Moreover, in spite of the broad variation in the relative concentration, the animals' secretions also present general related profiles in terms of protein contents, when analyzed by SDS-PAGE (Figure 4). Furthermore, the RP-HPLC secretion profile does not change although it is fresh skin secretion collected *in situ*, fresh skin secretion of animals kept in the animal facilities for years, or long stored skin secretion that was collected *in situ* and dried/crystallized by plain evaporation (data not shown). These features oppose what was described for dendrobates and the dependence of the skin secretion contents on the diet [25].

More specifically, regarding the three alkaloids found in *R. guttatus* skin secretion, there was only one identified in *R. major* secretion. However, both animals share the same habitat (Amazon forest) and diet. Nevertheless, they present significantly different alkaloid content. The same phenomenon can be observed for the steroidal secretion. For example, *R. major* skin secretion solution contains the bufadienolide steroids hellebrigenin and telocinobufagin found in several toads, whereas *R. guttatus* secretion contained sole one different steroid: resinobufagin, though animals share the habitat. Although some works [26] describe the possibility that, for *Melanophryniscus* genus, the alkaloids present in the skin would come exclusively from the diet (ants, mites, beetles, and millipedes), this is not the case for the *Rhinella* and *Rhaebo* studied here, for the difference in the skin secretion contents described in this work was observed for animals that live in the same habitat and share the same diet. Moreover, skin secretion contents, in terms of their C18 RP-HPLC and SDS-PAGE profiles, were not affected whether the animals had been just captured or maintained for periods of several months in captivity (data not shown).

Another factor not affecting the skin secretion contents was morphology: all toads have marinobufagin and just *R. major* does not have telocinobufagin in its skin secretion. Despite they live in completely different habitats and occupy different evolutionary positions and, more evidently, they have pronounced morphological differences, compared to the size of their bodies, *R. jimi* and *R. icterica* have protuberant and large parotoid glands whereas *R. granulosa* paratoid glands are very small and discrete.

On the other hand, *R. marina* and *R. major, *animals in different position on the phylogenetic tree, but collected at the same environment, showed some molecules in common but others were very different: while *R. major* has hellebrigenin, hellebrigenol-3-O-sulfate, and N′-N′-dimethyl-serotonin, *R. marina* has bufalin and marinobufagin. In this case, the environment they inhabit and their diet do not seem to be important for the composition of the parotoid secretion.

Authors have described alkaloids and steroids as toxic and antifeedant agents, acting as a major chemical defense strategy against predators [27, 28]. Such molecules act on the cardiovascular system, raising the blood pressure and/or increasing the contraction force of the heart [5, 8]. The protein contents, on the other hand, do not seem to be an important factor for toads' defense, since no significant or evident variation could be observed among the animals, suggesting that proteins might not function as toxins, but may be basically used as homeostatic/housekeeping agents. For instance, in other amphibian genera (*Phyllomedusa* [29] and *Leptodactylus* [30]) the protein contents in their skin secretions seem to have evolved towards being less representative, which may be an indication that proteins were not selected as major cutaneous toxins. One classical example would be the species of *Phyllomedusa* that have evolved many different peptides as their main (almost exclusive) cutaneous toxins [29, 31–36].

According to Clarke [27], “the ability of amphibians to survive in such a broad diversity of habitat types may be attributed to the evolution of many different morphological, physiological, biochemical and behavioral adaptations.” Moreover, the author believes that “the biochemical elaboration inherent in amphibian skin function is necessarily complex given the variety of roles and the range of chemical compounds involved together with the requirement for their unimpaired simultaneous functioning.”

Therefore, classical skin secretion comparison, for example, by means of the protein contents as assessed by SDS-PAGE may lead to false assumptions in terms of the similarity among amphibian poisons. This work clearly shows that the analysis of Figure 4 (SDS-PAGE), in spite of some minor differences in the total protein concentration and relative concentration within the same sample, would lead to the conclusion that the secretions are very similar among themselves. On the other hand, if one considers only the analyses performed by LC-MS (data not show), most likely the conclusion would be that these animals secrete in their parotoids different substances. It is only when one analyzes Figure 1 that a glimpse of a standardized (e.g., consistent, constant) variation arises. Initially, there is a distinct two-group peak distribution along the profile: alkaloids (14–17′) and steroids (20–26′), that is, constant for all animals. Next, one can observe that alkaloids always correspond to the largest peaks, regardless of their identity, and that these peaks are, approximately, two times larger than the steroids peaks. Therefore, there is indeed variation. However, the variation pattern is standardized. Hence, it is not only the apparent steadiness of the proteins in the SDS-PAGE analyses or the very complex profiles in LC-MS that represent the toxin contents and variation pattern present in these animals it is the sum of the variations of all features that must be taken into account when one intends to understand the variation trends molecules secreted by amphibian skin.

A recent work of Rash et al. [37] corroborates this hypothesis by showing that *Rhinella marina* skin secretion does contain peptides, although in such low level that, according to the authors, they are not toxins and may only represent the breakdown of structural proteins. Nevertheless, the presence of such peptides, derived from known proteins but presenting functions other than the nonclassical precursors (now known as cryptides), may represent the beginning of the trend for peptide secretion in the skin. Whether cryptides are the original bioactive peptides or they represent a local alternative to a given situation has already been reviewed by Pimenta and Lebrun [38]. Further studies are necessary to assess whether *R. marina* peptides are cryptides, for example, whether they possess biological activities. Moreover, our LC-MS analyses performed on *Rhinella's* secretions in this study do present a few *m/z* values that are compatible with peptides, both for the parent ion and for the daughter ions (data not shown); however, due to their low abundance no unambiguous identification could be made in this work. 

 In sum, no one single feature is sufficient to establish a thorough comparison among species. Even though this paper describes the standardized variation in the alkaloid and steroid contents of the skin secretion solution of nine different toads, it took other features (literature included) in order to suggest that there is a change trend in composition of the skin secretion of the Amphibia, for example, from a viscous alkaloid/steroid/protein rich parotoid secretion (in the Brazilian toads) to a clean and clear “warehouse” of peptides, in the case of *Phyllomedusa*.

## Figures and Tables

**Table 1:** Species used in this work.

Species	Habitat	Group
*Rhaebo guttatus *	Amazon Rainforest	*Rhaebo guttatus *

*Rhinella jimi *	Semiarid (Caatinga)	*Rhinella marina *
*Rhinella icterica *	Atlantic Rainforest	

*Rhinella major *	Amazon Rainforest	*Rhinella crucifer *
*Rhinella granulosa *	Semiarid (Caatinga)	
*Rhinella crucifer *	Atlantic Rainforest	

*Rhinella schneideri *	Savanna (Cerrado)	*Rhinella marina *
*Rhinella marina *	Amazon Rainforest	

*Rhinella margaritifera *	Amazon Rainforest	*Rhinella margaritifera *
